# Using YouTube to Learn Anatomy: Perspectives of Jordanian Medical Students

**DOI:** 10.1155/2020/6861416

**Published:** 2020-04-03

**Authors:** Ayman G. Mustafa, Nour R. Taha, Othman A. Alshboul, Mohammad Alsalem, Mohammed I. Malki

**Affiliations:** ^1^College of Medicine, QU Health, Qatar University, Doha, Qatar; ^2^Faculty of Medicine, Jordan University of Science and Technology, Irbid, Jordan; ^3^Faculty of Medicine, Jordan University, Amman, Jordan

## Abstract

**Purpose:**

The study is aimed at exploring the popularity, impact, and usefulness of using YouTube in learning anatomy as perceived by Jordanian medical students studying at Jordan University of Science and Technology.

**Methods:**

The present work is a cross-sectional questionnaire-based study. First-, second-, and third-year medical students were invited to complete an anonymous questionnaire. Students' responses were numerically coded, and the results were analyzed to reveal any statistically significant differences related to gender or level of study.

**Results:**

The results showed that 96.4% of the students used YouTube in general, 91.2% used it as a source of information, and 83.9% used YouTube as a learning tool in medical school. Further, YouTube was used by 79.1% of the students as an anatomy-learning tool. Most of these students used this platform in learning gross anatomy. The study also revealed that dissection videos were the most viewed anatomy-related content. Regarding the perceived value of YouTube as an anatomy-learning tool, the majority of the students reported that YouTube offered them useful anatomical information and enhanced their understanding, memorization, and recall of anatomical information. In addition, most of them recommended using YouTube as an anatomy-learning tool. Statistical analysis of the results revealed the presence of gender-related significant differences in students' perspectives. Such differences were also found among students of different levels of study.

**Conclusion:**

Medical students have positive attitudes toward using YouTube in augmenting their anatomy learning. For this, educators are encouraged to adopt YouTube as an educational tool in their anatomy instruction and to create new anatomy-related YouTube videos to enhance their students' learning.

## 1. Introduction

As a science that studies the structure of the human body, anatomy is considered a keystone in medical education. It stands to reason that medical students should have a solid anatomical knowledge of the body they will be dealing with in their future medical practice. Such anatomical knowledge is fundamental for physical examination, symptom interpretation, surgical procedures, and dealing with sophisticated imaging techniques used for three-dimensional visualization [1, 2]. Based on this, anatomy instructors should spare no effort in enhancing their students' learning. Traditionally, anatomy education had been dependent on dissection. However, the role of this teaching method is undergoing an obvious decline, especially with the emergence of various interactive technology-based educational tools. Students of the “Net Generation,” “YouTube Generation,” “ Generation Connected”, “Millennials,” or “Digital natives,” as they are termed, have their own attitudes, expectations, and learning styles [3–6]. Pedagogically accommodating these learning styles and preferences is central for achieving an optimal educational outcome. Indeed, utilization of computer- and web-assisted techniques in teaching these digital native students represents a quantum leap in anatomy education. Various studies reported a positive impact of implementing computer- and web-based activities into a gross anatomy curriculum [7–12].

Furthermore, a paradigm shift toward the use of web 2 and user-generated content has been evident in the last years. Social media websites including Facebook, YouTube, Twitter, Flickr, and Instagram are good examples. Because of their popularity, availability, and users' acceptance, several studies postulated that a beneficial educational role could be played by such websites [13–15]. For example, a study conducted at the University of Sharjah found that a faculty-operated Facebook Page was effective in supplementing anatomy education beyond the traditional classroom lectures [16]. Another study that was done in the University of Southampton found that incorporating Twitter as a way of supporting students' learning in neuroanatomy module had a positive impact on medical students [17]. Given the fact that anatomy is a visual science, it is expected that learning anatomy is enhanced by using visual methods, including videos and other immersive technologies that increase visual engagement. As previously reported, with a well-designed video, anatomical footage of cadavers and live patients, medical imaging, plastic models, and diagrams can be utilized to maximize the understanding of 3D structures through the 2D medium of the video [18].

YouTube is a video-sharing website that was officially launched in 2005. Via this platform, users can create their own channels, view, upload, and share video clips at no cost. In addition, people use this platform to search for information, participate in discussions, watch the news, and keep up with current issues. YouTube is a popular source of information among the students of the Net generation. Besides the fact that YouTube videos provide students with instant information at no cost, using these videos is reported to boost student's engagement, enhance their perception of learning efficacy, promote critical thinking, and deepen their understanding and visualization of the material [19–21]. A large and continuously increasing number of anatomy-related video clips are available on YouTube. This anatomy-related content includes a wide spectrum of animations, dissection videos, recorded lectures, radiological anatomy tutorials, clinically oriented demonstrations, and much more. A study that was conducted in Dublin college reported that the vast majority of surveyed second-year medical and radiation therapy students had used web-based platforms to source information with 78% using YouTube as their primary source of anatomy-related video clips [6]. This comes in harmony with the findings of another study which surveyed Venezuelan first-year medical students and found that 85% used YouTube videos to study human anatomy [22]. Both of the two aforementioned studies suggested a beneficial effect of implementing YouTube videos in anatomy education. Indeed, using a faculty-created anatomy education channel helped in learning anatomy as reported by the majority of second-year medical students at the University of Sharjah [23]. Such reports suggest that efficient deployment of YouTube videos, as a student learning aid, might be helpful in building contemporary anatomical knowledge that is essential for clinical practice.

The hypothesis made by the authors is that Jordanian medical students recognize the virtues of YouTube as a source of information and as an educational tool and they are using it to learn anatomical sciences. Therefore, this study is designed to test the aforementioned hypothesis both quantitatively and qualitatively. The study does so by reporting the prevalence of using YouTube in learning various anatomical sciences among Jordanian medical students. Moreover, the study reports the perspectives and attitudes of Jordanian medical students toward their experience with using YouTube as a learning tool of anatomy.

## 2. Materials and Methods

The present work is a cross-sectional, questionnaire-based study exploring YouTube as an anatomy-learning tool among medical students studying at JUST.

### 2.1. Questionnaire Design

The questionnaire consisted of four sections. The first one included questions to identify the demographic data of the participants who were asked to report their gender, age, nationality, and level of study. The second section consisted of four Yes/No questions that are aimed at gauging the general, informational, and medical- and anatomy-related educational usage of YouTube among the participants. The last two sections targeted only participants who used YouTube in learning anatomy. The third section included four multiple dichotomous questions by which participants were asked to choose the anatomical sciences, systems, and regions for which they used YouTube to learn. In addition to that, students were also asked to report the type of anatomy-related content they viewed on this website. The last section consisted of six Yes/No questions that focused on evaluating the value of YouTube in anatomy learning as perceived by the participants. Finally, participants were asked whether they have been advised by their instructors to use this platform in learning anatomy and whether they are willing to advise other students to use it.

### 2.2. Data Collection

A paper-based anonymous questionnaire was manually delivered to and collected from first-, second-, and third-year medical students who were willing to participate in the study. Five hundred questionnaires were distributed to medical students. Participation was voluntary with no incentives offered. Questionnaire administration was done during practical lab sessions of anatomy courses offered to medical students at JUST.

### 2.3. Data Processing and Analysis

A total of 411 completely answered questionnaires were obtained. The collected data were numerically coded for statistical analysis. A positive answer was given code one, and a negative answer was coded zero. To reveal any statistical differences related to gender and/or level of study, Pearson's Chi-square test was used. Differences were considered significant whenever the *P* value was less than 0.05 (*P* < 0.05). The statistical analysis was performed using the Statistical Package for Social Sciences software (SPSS, Inc., Chicago, IL).

## 3. Results

### 3.1. Background of the Participants

Section 1 of the questionnaire is aimed at identifying the demographic data of the participants including age, gender, nationality, and level of study. All students were Jordanians with an age range of 17–21 years, distributed as follows: 51.8% of males (*n* = 213), 48.2% females (*n* = 198), 22.8% first-year students (*n* = 94), 34.1% second-year students (*n* = 140), and 43.1% third-year students (*n* = 177).

### 3.2. Educational and Noneducational YouTube Usage

Section 2 of the questionnaire is aimed at gauging the general, informational, and medical- and anatomy-related educational usage of YouTube among the students. The results of this section in relation to gender and/or level of study are illustrated in Table 1.

Statistical analysis of students' responses to this section showed the following results:

(1) There were no significant differences related to gender
(2) Differences among students of different levels of study were revealed as follows:
  - Significant in general, informational, and anatomy-related educational usage of YouTube (*P* < 0.05)
  - Highly significant in medical-related educational usage of YouTube (*P* < 0.001)

### 3.3. YouTube Usage in Learning Anatomical Sciences, Systems, and Regions

In section 3, students reported the anatomical sciences, systems, and regions they used YouTube to learn. Results in relation to gender and/or level of study are presented in Table 2.

Statistical analysis of students' responses showed the following results:

(a) Anatomical sciences
  1. YouTube was significantly used more by males in learning histology (*P* < 0.05)
  2. There were no significant differences related to the level of study
(b) Anatomical systems
  1. There were no significant differences related to gender
  2. There were highly significant differences (*P* < 0.001) among students of different levels of study
(c) Anatomical regions
  (1) There were no gender-related significant differences in using YouTube to learn the anatomy of the head and neck, thorax, abdomen, and limbs
  (2) Males used YouTube to learn the anatomy of the pelvis region significantly more than females (*P* < 0.05)
  (3) Differences in using YouTube to learn anatomical regions among students of different levels of study were as follows:
    - Highly significant in learning the anatomy of the head and neck, thorax, and limbs (*P* < 0.001)
    - Significant in learning the anatomy of the pelvis region (*P* < 0.05)
    - Not significant in learning the anatomy of the abdomen

### 3.4. Viewed YouTube Anatomy-Related Content

Also in section 3, students reported the type of anatomy-related material they found on YouTube. Results in relation to gender and/or level of study are presented in Table 3.

Statistical analysis of students' responses showed the following results:

(1) Females viewed videos of anatomical images significantly more than males (*P* < 0.05)
(2) Differences in the type of viewed anatomy-related content among students of different levels of study were as follows:
  - Significant in viewing surgical operations and histological slides (*P* < 0.05)
  - Highly significant in viewing clinical cases (*P* < 0.001)

### 3.5. Perceived Value of YouTube as an Anatomy Learning Tool

Section 4 explored students' perceived value of using YouTube as an anatomy learning tool. In addition, the students reported whether they were advised to use YouTube in learning anatomy by their instructors and whether they recommend using it. Results in relation to gender and/or level of study are presented in Table 4.

Statistical analysis of students' responses showed the following results:

1. Female students perceived using YouTube to be helpful in memorization and recall of anatomical information significantly more than male students (*P* < 0.05)
2. There were highly significant differences among first-, second-, and third-year students who reported being advised by their instructors to use YouTube in learning anatomy (*P* < 0.001)

## 4. Discussion

In harmony with previously published studies [24–27], our results substantiate the broad popularity of YouTube as an online video platform. Actually, the remarkably high percentage of students who reported using YouTube assures that the term “YouTube Generation” is well-earned. In addition to the widespread usage of YouTube among the students, a vast majority of them (91.2%) reported using it as a source of information. This emphasizes that when dealing with a generation of students who are usually described as being “born with a chip” [28], one should consider their decidedly different preferences and predilections compared to formative generations. A student who grew up in a technological environment, surrounded by computers, laptops, smartphones, and iPads, is certainly very different from one whose books at the library were his/her main educational resource. Thus, one can predict that a traditional, old-fashioned lecture in which the presenter relies on using a chalkboard, for example, is most probably far from the expectations of these digital native students. So, based on the results of this study and many previous ones [6, 13–15, 23, 28–31], the available social media including YouTube can seemingly offer an advantageous educational value that should be utilized in creating a preferable educational environment.

Medical students of the “Net Generation” are avowedly keen to use internet resources in their learning. In accord with a previous study where 98% of medical students reported using YouTube as a source of acquiring medical knowledge [23], a majority of our students (83.9%) reported using YouTube as a learning tool in medical school, indicating that using this platform is apparently favored by medical students.

Moreover, a majority of the students reported using YouTube specifically in learning anatomy. While 92.9% of these students used YouTube to learn gross anatomy, only 25.8% of them used it in learning histology. This might be explained by the fact that although both are visual sciences, more three-dimensional visualization is needed to build a sound knowledge of gross anatomy. In contrast, histological knowledge is more dependent on visualization of two-dimensional histological slides. Thus, one can assume that labeled images, true histological slides, and virtual microscopy are reasonably more valuable than videos in learning histology. In addition, the results showed that 34.2% of YouTube-anatomy learners used it in learning embryology. Indeed, embryology is an anatomical science that demands visualization of the simultaneous stages of embryological development, which might be challenging for students. And so, its learning is expected to be enhanced by using visual tools such as YouTube videos. Embryology is taught to medical students at JUST through dedicated lectures within gross anatomy courses and system-based modules. Thus, the percentage of students using YouTube to learn embryology would possibly be higher than reported in this study if embryology was taught to medical students through a separate course.

Further, students appear to use YouTube more often in learning certain anatomical systems and regions. This might reflect the difficulty of learning these topics as perceived by the students and, accordingly, the need for using additional educational resources including YouTube videos to learn the anatomy of these systems and regions.

Dissection videos were the most viewed anatomy-related YouTube content. This is in harmony with previous work reporting that dissection videos are well received by students [32, 33]. A general positive attitude toward such videos may be present, and since many universities including JUST have abandoned cadaver dissection, our results postulate that dissection videos can be used as an alternative. Moreover, lower percentages of students viewed clinically oriented anatomical content including surgical operations, clinical cases, and radiographs indicating that medical students at JUST mostly use YouTube to learn basic rather than clinically oriented anatomy.

Students seem to have a very positive attitude toward YouTube as an anatomy-learning tool. This is manifested by high percentages of students reporting that they found useful anatomy-related information on YouTube and that using such information helped them in understanding, memorizing, and recalling anatomical information. As perceived by students, a successful anatomy learning involves various combinations of memorization, understanding, and visualization [34]. Actually, videos are suggested to have a positive impact on these mental processes. This suggestion is fortified by the results of a study in which videos uploaded to an anatomy YouTube channel were reported to be helpful in creating memorable visual images [23]. Various studies investigated the impact of gender on learning modalities and preferences. While some suggested the presence of gender-related differences in learning styles and preferred methods of information delivery among medical students [35, 36], other studies found no significant differences [37, 38]. Our results revealed gender-related significant differences in using YouTube to learn histology, anatomy of the pelvis region, and in viewing image-based videos. In contrary to a previous study which reported that gender has no impact on the perceived value of YouTube in teaching and learning anatomy [19], a significantly higher percentage of females reported that YouTube helped them in memorizing and recalling anatomical information. A plausible reason for this could be related to gender-related differences in spatial ability and mental rotation skills which are believed to be beneficial for learning anatomy [39]. Interestingly, previous studies reported the presence of gender-related differences among medical students in terms of generic spatial ability [40] and anatomy mental rotation tests [41]. So female Jordanian medical students might be relying on YouTube videos to boost their three-dimensional visualization of anatomical structures and enhance their mental rotation skills leading to a better memorable understanding. Accordingly, females probably perceive the educational value of such videos in improving memorization and recall of anatomical information more.

The results also revealed the presence of significant differences in general, informational, and medical- and anatomy- related educational use of YouTube among students of different levels of study. In addition, such differences exist in the anatomical systems and regions that students used YouTube to learn. Several suggested factors may stand behind these findings; one might be the fact that students of different years are taught by different instructors who adopt different pedagogical methods. This point can be substantiated by the presence of highly significant differences among students of different levels who reported being advised to use YouTube by their instructors. Another factor might be simply related to the differences in anatomy curricula offered to different levels of study at JUST. Moreover, as medical students go through their preclinical education, they gradually become more clinically oriented in their learning. This is manifested by the higher percentages of third-year medical students who viewed anatomy-related clinical cases and surgical operations found on YouTube.

Lastly, although only 68% of students were advised by their instructors to use YouTube in anatomy learning, 94.5% were willing to advise their colleagues to use this platform. Indeed, the latter percentage reflects an excellent learners' satisfaction with YouTube as an anatomy-learning tool. However, a noteworthy limitation of using YouTube in education is the absence of adequate quality regulation. Indeed, YouTube anatomy-related videos are of variable quality and are accordingly of a variable educational value. For example, in a study that evaluated the usefulness of YouTube videos in learning surface anatomy, it was reported that some areas were covered by useful educational videos such as the surface anatomy of the shoulder, knee, leg, ankle, carotid artery, and dermatomes [42]. Nonetheless, as reported by the same study, other areas were either poorly covered or not covered at all, and thus, YouTube was considered an inadequate source of surface anatomy information. In a similar vein, another study [43] screened human heart anatomy videos and reported that anatomy of the atria, ventricles, and the great vessels was well covered in YouTube, but the position of the heart and its relation to adjacent viscera were poorly covered. In fact, searching for appropriate, reliable, and educationally effective YouTube videos might be challenging and time consuming for students. To overcome this, educators are encouraged to create anatomy YouTube channels and to provide their students with suggested links that meet the course objectives and match the students' level of knowledge.

## 5. Conclusion

YouTube has the potential to play a valuable role in sowing the seeds of a fruitful anatomical knowledge that can be successfully applied in clinical practice. Medical students have positive attitudes toward using this platform in augmenting their anatomy learning, and as perceived by the majority of them, YouTube videos can potentiate understanding, memorization, and recall of anatomical information leading to a good learners' satisfaction. Furthermore, gender differences may have a role in this perceived value.

Rather than standing still when the winds of change are blowing, educators are encouraged to adopt the emerging educational tools including YouTube. This tool has the power to captivate digital native students and enhance their anatomy learning. Since the available YouTube anatomy-related content is deemed inadequate, the responsibility of creating new content, that fully exploits the potential offered by YouTube, is placed upon anatomy educators. In fact, anatomy educators should never be reluctant to update their teaching skills so that they can ignite their students' interest in learning anatomy, making their anatomy courses beneficial, enjoyable, and memorable.

Although this study has been informative about the use of YouTube in learning anatomy, it has some limitations. The questionnaire was general and exploratory in nature. For example, it did not include questions that evaluate students' satisfaction with YouTube videos for specific anatomic subjects. Further research is needed to outline all possible applications of YouTube before integrating it as a teaching method in anatomy curricula. In addition, the proposed usefulness of creating anatomy YouTube channels and its effect on Jordanian medical students' satisfaction with YouTube as an educational tool has to be tested. Another suggested future direction is to allow medical students themselves to create anatomy-related YouTube videos and to evaluate if this can boost their retention of anatomical knowledge. Moreover, perspectives of anatomy educators on using this platform as a pedagogical method need to be also investigated.

### 5.1. Practice Points

- Social media websites including YouTube can be harnessed as a potent educational tool because of their availability, popularity, and acceptance by the students.
- YouTube is used by a majority of students as an anatomy learning tool.
- Students find YouTube videos useful and helpful in understanding, memorization, and recall of anatomical information.
- Educators are encouraged to use YouTube in anatomy instruction and to provide their students with anatomy channels and suggested links for useful and educationally effective anatomy-related videos.

## Figures and Tables

**Table 1:** Measuring general, informational, and medical- and anatomy-related YouTube usage among medical students in relation to gender and level of study.

Question	% + ve	M	F	*P* value	1^st^	2^nd^	3^rd^	*P* value
Do you use YouTube in general	96.4	97.7	94.9	.144	91.5	99.3	96.6	.008
Do you use YouTube as a source of information	91.2	91.5	90.9	.819	85.1	94.3	92.1	.045
Do you use YouTube as a learning tool in medical school	83.9	82.6	85.4	.452	70.2	90.0	86.4	.000
Do you use YouTube to learn anatomy	79.1	75.6	82.8	.071	67	79.3	85.3	.002

% + ve: percentage of positive answers; M: males; F: females; 1^st^: first-year students; 2^nd^: second-year students; 3^rd^: third-year students.

**Table 2:** Using YouTube in learning anatomical sciences, systems, and regions in relation to gender and level of study.

Using YouTube in learning:	% + ve	M	F	*P* value	1^st^	2^nd^	3^rd^	*P* value
Gross anatomy	92.9	91.9	93.9	.487	95.2	88.3	95.4	.064
Histology	25.8	31.1	20.7	.034	30.2	23.4	25.8	.621
Embryology	34.2	32.9	35.4	.642	23.8	34.2	38.4	.122
Nervous system	46.2	44.1	48.2	.462	12.7	10.8	86.1	.000
Cardiovascular system	52.3	50.9	53.7	.623	42.9	69.4	43.7	.000
Musculoskeletal system	55.1	50.9	59.1	.137	71.4	30.6	66.2	.000
Urogenital system	14.2	16.1	12.2	.307	27	3.6	16.6	.000
Gastrointestinal system	31.7	32.9	30.5	.638	42.9	9	43.7	.000
Respiratory system	41.8	42.2	41.5	.888	44.4	59.5	27.8	.000
Anatomy of the head & neck	58.2	57.1	59.1	.714	71.4	41.4	64.9	.000
Anatomy of the thorax	43.7	46	41.5	.414	42.9	60.4	31.8	.000
Anatomy of the abdomen	38.5	37.3	39.6	.661	41.3	34.2	40.4	.525
Anatomy of the pelvis	23.1	28	18.3	.039	34.9	13.5	25.2	.004
Anatomy of the limbs	46.8	43.5	50	.239	47.6	28.8	59.6	.000

% + ve: percentage of positive answers; M: males; F: females; 1^st^: first-year students; 2^nd^: second-year students; 3^rd^: third-year students.

**Table 3:** YouTube anatomy-related content viewed by students in relation to gender and level of study.

Viewed YouTube anatomy-related content	% + ve	M	F	*P* value	1^st^ year	2^nd^ year	3^rd^ year	*P* value
Dissection videos	82.5	85.1	79.9	.216	82.5	79.3	84.8	.513
Cadaveric sections	30.2	29.2	31.1	.708	20.6	31.5	33.1	.179
Surgical operations	38.8	38.5	39.0	.924	20.6	36.9	47.7	.001
Images	41.5	35.4	47.6	.026	49.2	44.1	36.4	.177
Histological slides	23.1	24.2	22.0	.627	17.5	31.5	19.2	.032
Study tips and advice	13.5	12.4	14.6	.560	19.0	13.5	11.3	.316
Radiographs	9.5	11.8	7.3	.169	6.3	14.4	7.3	.096
Clinical cases	19.1	19.3	18.9	.936	6.3	14.4	27.8	.000
Lectures	34.8	33.5	36.0	.645	28.6	36.0	36.4	.515

% + ve: percentage of positive answers; M: males; F: females; 1^st^: first-year students; 2^nd^: second-year students; 3^rd^: third-year students.

**Table 4:** Perceived value of YouTube as an anatomy-learning tool in relation to gender.

Question	% + ve	M	F	*P* value	1^st^	2^nd^	3^rd^	*P* value
Have you found any useful information relevant to anatomy on YouTube	96.9	96.9	97.0	.976	93.7	97.3	98.0	.233
Has using YouTube helped you to understand any anatomical topics	96.6	95.0	98.2	.118	93.7	97.3	97.4	.350
Has using YouTube helped you to memorize and recall anatomical information	89.2	85.7	92.7	.043	87.3	88.3	90.7	.705
Has using YouTube helped you to get higher marks in anatomy exams	48.9	49.1	48.8	.959	46.0	55.0	98.0	.293
Has any of your instructors in the anatomy department advised you to use YouTube in learning anatomy	68.0	64.0	72.0	.123	90.5	22.5	97.4	.000
Do you advise other students to use YouTube as a learning tool for anatomy	94.5	93.2	95.7	.312	92.1	96.4	90.7	.463

% + ve: percentage of positive answers; M: males; F: females; 1^st^: first-year students; 2^nd^: second-year students; 3^rd^: third-year students.

## Data Availability

The datasets used and/or analyzed during the current study are available from the corresponding author on reasonable request.
